# An Economical Composite Membrane with High Ion Selectivity for Vanadium Flow Batteries

**DOI:** 10.3390/membranes13030272

**Published:** 2023-02-24

**Authors:** Yue Zhang, Denghua Zhang, Chao Luan, Yifan Zhang, Wenjie Yu, Jianguo Liu, Chuanwei Yan

**Affiliations:** 1 Institute of Metal Research, Chinese Academy of Sciences, Shenyang 110016, China; 2 School of Materials Science and Engineering, University of Science and Technology of China, Hefei 230026, China; 3 College of Chemistry, Liaoning University, Shenyang 110036, China

**Keywords:** vanadium flow batteries, low cost, thin composite membrane, polyethylene, perfluorosulfonic acid

## Abstract

The ion exchange membrane of the Nafion series widely used in vanadium flow batteries (VFBs) is characterized by its high cost and high vanadium permeability, which limit the further commercialization of VFBs. Herein, a thin composite membrane enabled by a low-cost microporous polyethylene (PE) substrate and perfluorosulfonic acid (PFSA) resin is proposed to reduce the cost of the membrane. Meanwhile, the rigid PE substrate limits the swelling of the composite membrane, which effectively reduces the penetration of vanadium ions and improves the ion selectivity of the composite membrane. Benefiting from such a rational design, a VFB assembled with the PE/PFSA composite membrane exhibited a higher coulombic efficiency (CE ≈ 96.8%) compared with commercial Nafion212 at 200 mA cm^−2^. Significantly, the energy efficiency maintained stability within 200 cycles with a slow decay rate. In practical terms, the thin PE/PFSA composite membrane with low cost and high ion selectivity can make an ideal membrane candidate in VFBs.

## 1. Introduction

With the development of society and the increase in population, the consumption of fossil energy is accelerating. In order to achieve sustainable development, the scale of renewable energy utilization is beginning to expand rapidly [1]. Due to the intermittent and volatile nature of renewable energy, large-scale energy storage systems are being vigorously developed. Among them, the vanadium flow battery (VFB) has good prospects, benefiting from unique features such as a flexible design, long service life, and rapid response, especially high-level reliability and safety [2,3]. Among the components of the VFB, the proton exchange membrane is crucial; it plays the role of conducting protons and hindering the migration of positive and negative redox-active substances [4,5]. The membrane must ensure the VFB possesses high efficiency, excellent long-term cycling, and low operating cost.

In the field of proton exchange membranes used in VFBs, perfluorosulfonic acid (PFSA) membranes are widely used due to their high proton conductivity and outstanding chemical stability [6,7,8]. However, these membranes have a high production cost, meaning they account for about 40% of the total cost of the cell [9,10]. In addition, the poor dimensional stability of hydrated PFSA membranes leads to serious vanadium ion penetration, resulting in low ion selectivity and low efficiency [11,12]. Therefore, PFSA membranes must be developed to have sufficient thickness to achieve better ion selectivity. Unfortunately, the thicker membranes induce high ohmic resistance and lead to low performance of the VFB [13,14]. At the same time, thickening them greatly increases the cost of the membranes and hinders the rapid engineering application of VFBs. As a consequence, the development of low-cost and high-performance membranes has become a research hotspot and the key to the industrialization of VFBs.

In order to overcome the shortcoming of the low ion selectivity of PFSA, various methods have been applied. Among those are the following: (a) introducing functional fillers such as aminated SiO_2_ [15], graphene oxide [16], g-C_3_N_4_ nanosheets [17], and lignin [7] as barriers in base resin to hinder vanadium ion transmission; (b) surface modification with substances such as a cationic charged polyethylenimine coating layer [18], PWA-CS multilayers [19], and NH_2_-POSS macromer [20]; and (c) adjusting the thickness and improving the pretreatment process of Nafion membranes [21,22]. These methods have proved to successfully reduce the mobility of vanadium ions, but cannot significantly reduce the total cost.

Additionally, introducing a porous substrate as a rigid support to enhance the ion selectivity is considered another effective option. Different porous substrates such as fluorocarbon polymers (polytetrafluoroethylene (PTFE)) and hydrocarbon polymers (polypropylene (PP), polyethylene (PE)) can be used for the fabrication of composite membranes due to their low cost and excellent chemical stability [23,24,25]. Moreover, given their excellent hydrophobicity, the incorporation of these substrates and PFSA resin will effectively improve the dimensional stability, leading to lower vanadium permeability and higher ion selectivity, which can support the requirements for VFBs [25,26,27]. Meanwhile, the mechanical strength of the composite membranes is greatly improved. However, the ion conductivity of the composite membranes inevitably decreases, which limits further applications. The thickness of these composite membranes usually exceeds 50 μm [26,28,29], and it seems it is possible to optimize the thickness of the composite membrane and the amount of PFSA resin to further reduce costs and improve performance [13].

In this study, to reduce the cost and optimize efficiency, we designed a thin composite membrane (~20 μm) composed of PFSA resin and low-cost PE porous substrate. The PE porous substrate was fully filled and wrapped on both sides by PFSA resin, as confirmed by SEM, and FTIR. Based on this combination, the required amount of the expensive ingredient, PFSA resin, was minimized, which reduced the overall cost. Meanwhile, the PE porous substrate could significantly increase the dimensional stability and ion selectivity of the composite membrane, which could ensure its performance. We investigated the mechanical and chemical properties of the composite membrane, then the vanadium ion permeability, electrochemical properties, and battery performance of the composite membrane were studied. The results provide a reference for the development of the next-generation composite membrane for VFBs.

## 2. Experimental Section

### 2.1. Materials

PFSA resin was purchased from Shandong Dongyue Company (Zibo, China). The PE substrate (12 μm) with a porosity of 42% was provided by Hebei Gellec New Energy Science & Technology Joint Stock Co., Ltd. (Handan, China) Nafion212 membrane was provided by Chemours (Wilmington, DE, USA). Graphite felt with a thickness of 4.4 mm was purchased from Liaoyang Jingu Material Co., Ltd. (Liaoyang, China) Phenolphthalein (98%) was purchased from Duksan Pharmaceutical Co., Ltd. (Ansan-si, Korea) N,N-dimethylformamide (DMF, ≥99%), concentrated sulfuric acid (H_2_SO_4_, 98%), sodium chloride (NaCl, 99.5%), and sodium hydroxide (NaOH, 98%) were provided by China National Pharmaceutical Group Co., Ltd. (Beijing, China) The chemicals were used as received, without further processing.

### 2.2. Preparation of the PE/PFSA Composite Membrane

**Figure 1:**
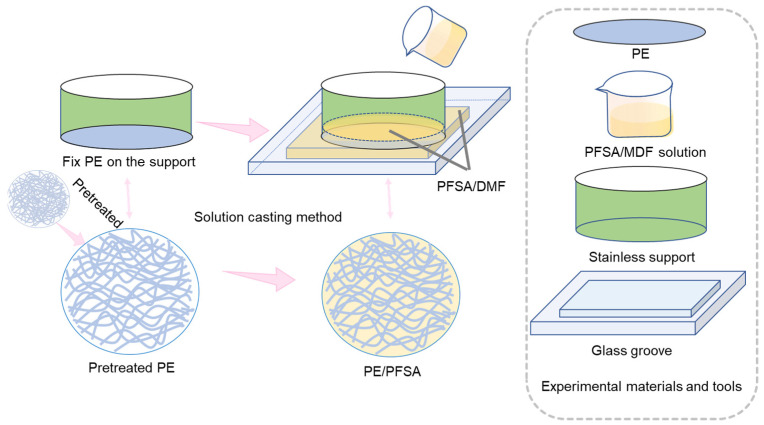
Schematic illustration of process for fabricating the PE/PFSA composite membrane.

Firstly, stoichiometric PFSA was added to DMF to dilute it to a homogenous 1 wt% PFSA solution, and the prepared solution was stirred with an electromagnetic stirrer. Then, the PE substrate was first cut to the right size and next immersed in 3.0 M H_2_SO_4_ solution at 60 °C for 8 h. Afterward, the PE substrate was washed with deionized water several times and dried at 60 °C for 2 h. Later, the pretreated PE substrate was expanded by a circular stainless support with a diameter of 6 cm and immersed in PFSA solution in an ultrasonic instrument for 0.5 h. Next, the pretreated PE support was placed in a glass groove, and the prepared PFSA solution was poured into the glass groove and the inside of the support, then later dried at 100 °C overnight. Finally, the composite membrane was peeled off the glass groove. The final membrane was defined as PE/PFSA. For comparison, a pure PFSA membrane was also prepared in the same experimental condition. Importantly, the thickness of the two membranes was regulated at ~20 μm by controlling the volume of PFSA solution added (Figure 1).

### 2.3. Characterization Methods

A scanning electron microscope (SEM) (INSPECT-F, FEI, USA) was employed to characterize the superficial morphologies, including the surface, and a cross-section of the prepared membranes. All samples were tested at a working voltage of 10 kV and a detecting distance of 10 mm. Meanwhile, an energy-dispersive X-ray spectrometer (EDS) was operated to detect the distribution of characteristic elements via mapping technology. The EDS images were acquired in 20 s and at a count rate of 10,000 counts per minute. The superficial structure was analyzed using a Fourier transform infrared spectrometer (FTIR) (IR Cary 630, Agilent Technologies, Santa Clara, CA, USA) at a wavelength of 400–4000 cm^−1^. We used the ATR experimental type of IR spectroscopy. The spectra were obtained with a resolution of 2 cm^−1^ and 64 cumulative detection times.

The water uptake (WU) and swelling ratio (SR) were calculated according to the changes in weight and dimension between wet and dry membranes. Firstly, the wet membranes were prepared by immersing the membranes in deionized water for 48 h and wiping off the residual water with filter paper. After that, the prepared wet membranes were weighted by an analytical balance and we measured the dimension values using a micrometer. Lastly, the weight and dimension values of the dry membranes were measured simultaneously as the corresponding parameters after the samples in a wet state were dried under a vacuum at 100 °C for 12 h. The WU and SR were calculated according to Equations (1) and (2):

(1) $$\mathrm{WU}\left(\%\right)=\frac{W_{w}-W_{d}}{W_{d}}\times 100\%$$

(2) $$\mathrm{SR}\left(\%\right)=\frac{X_{w}\cdot Y_{w}\cdot Z_{w}-X_{d}\cdot Y_{d}\cdot Z_{d}}{\begin{array}{c} X_{d}\cdot Y_{d}\cdot Z_{d} \end{array}}\times 100\%$$

where W represents the weight of the membrane; X, Y, and Z represent the length, width, and thickness of the membrane, respectively; and the subscripts w and d represent the wet and dry states of the membrane, respectively.

The ion exchange capacity (IEC) was determined using an acid-base titration method. The dried membranes were firstly immersed in 1 M NaCl solution for 48 h to completely replace the H^+^ of −SO_3_^−^ with Na^+^. Then, the solutions were titrated using a 0.01 M NaOH solution, and phenolphthalein as a pH indicator was used to judge whether the solution was neutral. The IEC was calculated according to Equation (3):

(3) $$\mathrm{IEC}=\frac{C_{\mathrm{NaOH}}\times V_{\mathrm{NaOH}}}{W_{d}}$$

where C_NaOH_ represents the molar concentration of NaOH solution, V_NaOH_ is the volume of NaOH solution used in the titration process, and W_d_ is the weight of the dry membrane.

The conductivity (σ) of membranes was calculated according to the parameters: area-specific resistance (ASR), and thickness. Firstly, the prepared membranes were soaked in 3.0 M H_2_SO_4_ for 1 d at room temperature primarily. Then, the immersed membranes were assembled in a conductivity cell. Following that, electrochemical impedance spectroscopy (EIS) was applied to assess the resistance of the conductivity cell with a membrane (R_2_) and without a membrane (R_1_) at an electrochemical station (Reference 600, Gamry, Warminster, PA, USA) with the frequency set to 100 kHz. The ASR and σ of the membranes were calculated based on Equations (4) and (5):

(4) $$\;\mathrm{ASR}=\left(R_{2}-R_{1}\right)\times S$$

(5) $$\sigma =\frac{L}{\begin{array}{c} ASR \end{array}\;}$$

where ASR represents the area-specific resistance, and S and L represent the actual test size (1.77 cm^−2^) of the conductivity cell and thickness of the membrane, respectively.

The permeability of VO^2+^ penetrating from the other side of the diffusion cell through the membranes was confirmed. The left and right sides of the diffusion containers comprised 100 mL 1.65 MgSO_4_ and 1.65 M VOSO_4_, both in 1.65 M VOSO_4_ solution. The solution was stirred at the speed of 300 rpm. The VO^2+^ concentration on the side of MgSO_4_ was estimated using an ultraviolet-visible (UV-vis) spectrophotometer (UN-1900, Beijing Puxi Instrument Co., Ltd., Beijing, China) 5 times every 24 h. The formula of VO^2+^ permeability (P) was as in Equation (6):

(6) $$\;P=\frac{L\times \;V}{S\times \left(C_{R}-C_{L}\left(t\right)\right)}\;\left(\frac{d\left(C_{L}\left(t\right)\right)}{\mathrm{dt}}\right)$$

where L, V, and S indicate thickness, the volume of solution in the container, and the effective area of the membrane, respectively. In addition, C_R_ and C_L_(t) represent the VO^2+^ concentrations in the right and left containers in real-time, respectively.

### 2.4. VFB Single-Cell Performance

The designed VFB single cell was assembled like a sandwich with a membrane (3 cm × 3 cm), two graphite felt electrodes (2.2 mm), and current collecting plates. The electrolyte containers were filled with 25 mL 1.65 M V^3+^/VO^2+^ (V^3+^/VO^2+^ = 1:1) in 3 M H_2_SO_4_ solution and the electrolytes were circulated through the single cell at a rate of 40 mL min^−1^. Then, the single cell was tested using a battery system (Neware CT-3008 −5 V/6 A) in the range of 100–300 mA cm^−2^, where the cut-off voltages were set as 1.65 V and 1 V. Accordingly, the coulombic efficiency (CE), voltage efficiency (VE), and energy efficiency (EE) were obtained based on the following formulas [30]:

(7) $$\mathrm{CE}=\frac{\int I_{d}\mathrm{dt}}{\int I_{c}\;\mathrm{dt}}\times 100\%$$

(8) $$\mathrm{EE}=\frac{\int V_{d}I_{d}\mathrm{dt}}{\int V_{c}I_{c}\mathrm{dt}}\times 100\%$$

(9) $$\mathrm{VE}=\frac{\mathrm{EE}}{\mathrm{CE}}\times 100\%\;$$

where V_c_ and V_d_ represent the voltages of the charging and discharging processes, respectively, and I_c_ and I_d_ are the currents. 

In addition, a life cycle test of the membranes was conducted at 200 mA cm^−2^, and the self-discharge performance was determined based on a 50% state of charge at 100 mA cm^−2^. When the voltage was less than 0.8 V, the self-discharge experiment was terminated.

## 3. Results and Discussion

**Figure 2:**
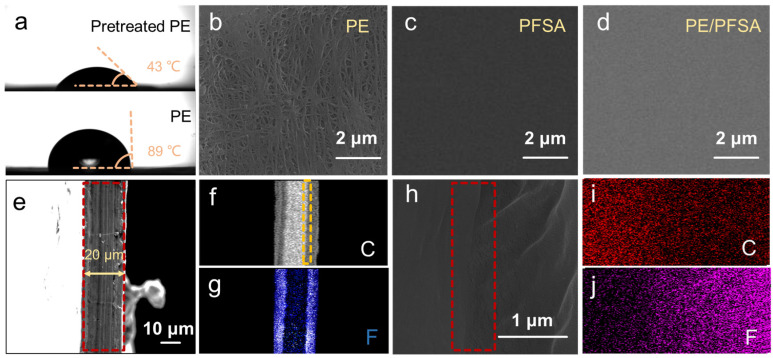
(**a**) Water contact angles of the original PE and the pretreated PE; surface morphologies of (**b**) PE, (**c**) pure PFSA, and (**d**) PE/PFSA; (**e**) cross-section morphology of PE/PFSA; (**f**,**g**) EDS mapping of (**e**) with C and F elements; (**h**) high-magnification SEM image of the interface in the selected area in Figure 2f; and (**i**,**j**) EDS mapping of (**h**) with C and F elements.

The contact angle test was carried out to explore the effect of sulfuric acid pretreatment on the hydrophilicity of the PE substrate. As shown in Figure 2a, after the pretreatment, the contact angle of the PE substrate was reduced from 89° to 43°. This indicates that the hydrophilicity of PE substrate was improved and PFSA solution could better infiltrate into the pores of PE.

SEM was used to observe the surface morphologies of PE, PFSA, and PE/PFSA membranes to evaluate the evolution of the surface state after PFSA resin infiltrated the PE substrate. From the SEM images, it can be seen that the PE substrate (Figure 2b) exhibited a typical dendritic structure and a rough surface state with many pores. The pore size was around 50~100 nm (Figure S2). Moreover, the surface of pure PFSA membrane (Figure 2c) displayed a smooth, dense, and homogeneous surface appearance without any defects. The morphology of the PE/PFSA composite membrane (Figure 2d) was also smooth and compact, similar to that of the pure PFSA membrane. This demonstrates that the pores of PE were fully filled by PFSA and the surface was successfully combined with PFSA. 

The cross-section morphology of the PE/PFSA membrane was ascertained to determine the combination state of PE and PFSA and the distribution of PFSA resin. As presented in Figure 2e, the thickness of the PE/PFSA membrane was 20 μm. Moreover, the cross-section revealed an integral and compact state with no defects. This further demonstrates that the PE substrate was sufficiently immersed, and the substrate and PFSA phase were nicely integrated. Furthermore, EDS mapping (Figure 2f,g) was carried out to verify the distribution of carbon (C) and fluorine (F) elements. The C element was concentrated in the middle of the composite membrane, confirming that PE substrate existed since PE substrate is mainly composed of C element. Moreover, the F element derived from the PFSA also appeared in the middle of the composite membrane, along with appearances on both sides. This indicates that the PFSA resin successfully infiltrated the PE substrate and PFSA covering layers were formed on both sides of the composite membrane. To further observe the binding state at the interface of PE and PFSA, high-magnification SEM was conducted. As shown in Figure 2h, at the interface, the morphology of the PE/PFSA composite membrane was still smooth and dense, without obvious delamination. This confirmed that PE and PFSA were well combined. The analysis of cross-section morphologies verified that a cross-linking network was successfully established in the PE/PFSA composite membrane and that the structure was dense.

**Figure 3:**
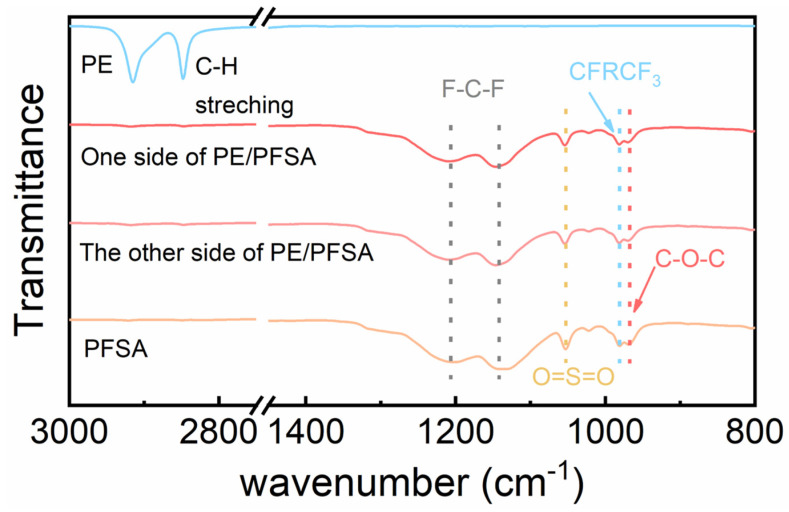
FTIR spectra of the PE, pure PFSA, and both sides of the PE/PFSA membrane.

The chemical structure of the PE/PFSA composite membrane at both sides was determined using FTIR spectroscopy. For comparison with the PE/PFSA composite membrane, the PE substrate and pure PFSA membrane were also measured by an FTIR test, as displayed in Figure 3. The characteristic bands of C-H stretching relevant to the bare PE substrate emerged at 2850–3000 cm^−1^ in wavenumber [31]. Notably, both sides of the PE/PFSA composite membrane show similar FTIR spectra to that of PFSA. The characteristic bands of F-C-F asymmetric and symmetric stretching could be seen at 1201 cm^−1^ and 1144 cm^−1^. In addition, the C-F stretching peak (-CF_2_-CF(CF_3_)- group) between the main chain and -SO_3_H occurred at 981 cm^−1^. Additionally, another two peaks at 1055 cm^−1^ and 968 cm^−1^ were attributed to O=S=O stretching and C-O-C stretching, respectively [32]. These results confirmed that both sides of the PE/PFSA composite membrane were covered by a PFSA layer, meaning the composite membrane was successfully prepared. 

**Figure 4:**
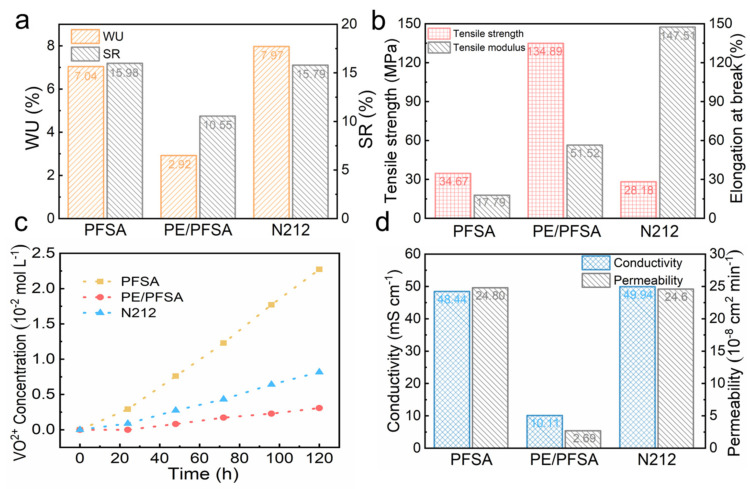
(**a**) Dimensional stability parameters, (**b**) mechanical performance parameters, (**c**) VO^2+^ concentration curve over time, and (**d**) proton conductivities and vanadium ion permeabilities of the membranes.

The physicochemical properties, including ion exchange capacity (IEC) and dimensional and mechanical stability, are important reference parameters of the membrane. As shown in Table S1, the IEC value of PE/PFSA (0.29 mmol g^−1^) was lower than those of Nafion212 (0.95 mmol g^−1^) and pure PFSA (0.90 mmol g^−1^). This was due to the introduction of PE without functional groups. Similarly, the PE/PFSA composite membrane exhibited the lowest swelling ratio (SR) and water uptake (WU) when compared with the pure PFSA and Nafion212 membranes. This equally resulted from the introduction of a rigid PE support and the restricted swelling of hydrophilic polymer PFSA in solution. In addition, the hydrophobic nature of PE substrate significantly reduced the water absorption capacity. Moreover, the mechanical properties of samples are shown in Figure 4b. The tensile strength of PE/PFSA was significantly higher than those of pure PFSA and Nafion212 due to the excellent mechanical properties of PE support. Its elongation at break was greatly improved compared with pure PFSA. Such composite membranes can well withstand the pressure induced by electrolyte flow, to ensure the stable operation of VFBs.

As a crucial parameter, a membrane’s VO^2+^ permeability accurately reflects its ability to inhibit vanadium ion penetration. Firstly, the variation of VO^2+^ concentration versus time is shown in Figure 4c. The pure PFSA membrane exhibits the maximum vanadium ion concentration due to its small thickness and weak dimensional stability. The PE/PFSA composite membrane shows the minimum vanadium ion concentration, much smaller than for pure PFSA and Nafion212. Meanwhile, the VO^2+^ ion permeability of PE/PFSA (2.69 × 10^−8^ cm^2^ min^−1^) is significantly lower than those of PFSA (24.8 × 10^−8^ cm^2^ min^−1^) and Nafion212 (24.6 × 10^−8^ cm^2^ min^−1^) (Figure 4d). This is because the denser and more compact structure of PE/PFSA hindered the permeation of vanadium ions. In addition, the composite membrane shows the lowest swelling ratio. That greatly reduced the expansion of the ion channel size of the composite membrane and inhibited the diffusion of vanadium ions. We also investigated the conductivity of the membranes, which reflected their ability to conduct protons. As expected, the conductivity of PE/PFSA inevitably decreased, as depicted in Figure 4d. The PE substrate’s insulation inevitably decreased the conductivity of the PE/PFSA composite membrane. In addition, dead-ends of PFSA conductive polymer in the PE substrate hindered proton transfer, which also affected ion conductivity. Combining the results, we can surmise that the ion selectivity of the composite membrane PE/PFSA (2.54 × 10^5^ S min cm^−3^) was higher than those of Nafion212 (1.97 × 10^5^ S min cm^−3^) and pure PFSA (2.01 × 10^5^ S min cm^−3^). Furthermore, since we introduced the PE substrate, the improved obstruction of VO^2+^ penetration compensated for the reduction in conductivity. Therefore, the ion selectivity relevant to the comprehensive performance of PE/PFSA was further improved.

**Figure 5:**
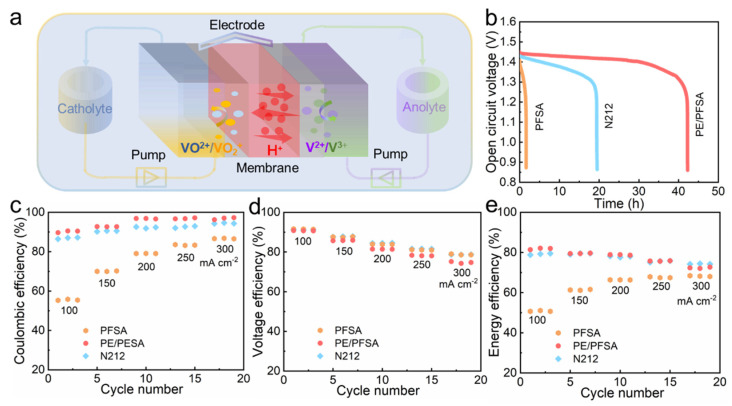
(**a**) Schematic illustration of operating principle for a VFB; (**b**) OCV curves over time at 100 mA cm^−2^; and (**c**–**e**) CE, VE, and EE of VFBs with pure PFSA, PE/PFSA composite, and Nafion212 membranes at current densities from 100 to 300 mA cm^−2^.

In order to determine the actual application performance of the composite membrane, membranes were assembled in the VFB single cell as shown in Figure 5a for charging and discharging test experiments. Firstly, we tested the persistent self-discharge performance of VFB with membranes characterized with the curve of open-circuit voltage (OCV). As exhibited in Figure 5b, the VFB equipped with PE/PFSA composite membrane demonstrated an outstanding performance where the OCV persisted for 42.2 h. Meanwhile, the duration of OCV beyond 0.8 V only reached 19.5 h and 1.6 h for the Nafion212 and pure PFSA membranes, respectively. This confirmed that the composite membrane restrained the permeation of vanadium ions due to the rigid constraint of PE. In addition, the coulombic efficiency (CE), voltage efficiency (VE), and energy efficiency (EE) of VFB assembled with these membranes were measured at current densities ranging from 100 to 300 mA cm^−2^. As shown in Figure 5c, the pure PFSA membrane exhibited extremely low CE at all current densities owing to its thinness and poor dimensional stability. Meanwhile, the PE/PFSA composite membrane manifested a higher CE, benefiting from the lowest vanadium ion diffusion rate. Moreover, the VE (Figure 5d) of the PE/PFSA composite membrane declined to a certain extent as predicted. Due to the introduction of the insulated substrate, the ion conductivity of the PE/PFSA composite membrane decreased and the ASR increased. This led to severe ohmic and voltage loss of the VFB cell, resulting in low VE. Most importantly, the parameter of EE combined with the influence of CE and VE reflected the ability of VFB cells to convert energy. As illustrated in Figure 5e, the VFB with the PE/PFSA composite membrane demonstrated a high EE value of 79.8% at 200 mA cm^−2^, which was comparable to that of Nafion212 and significantly higher than that of the pure PFSA membrane.

**Figure 6:**
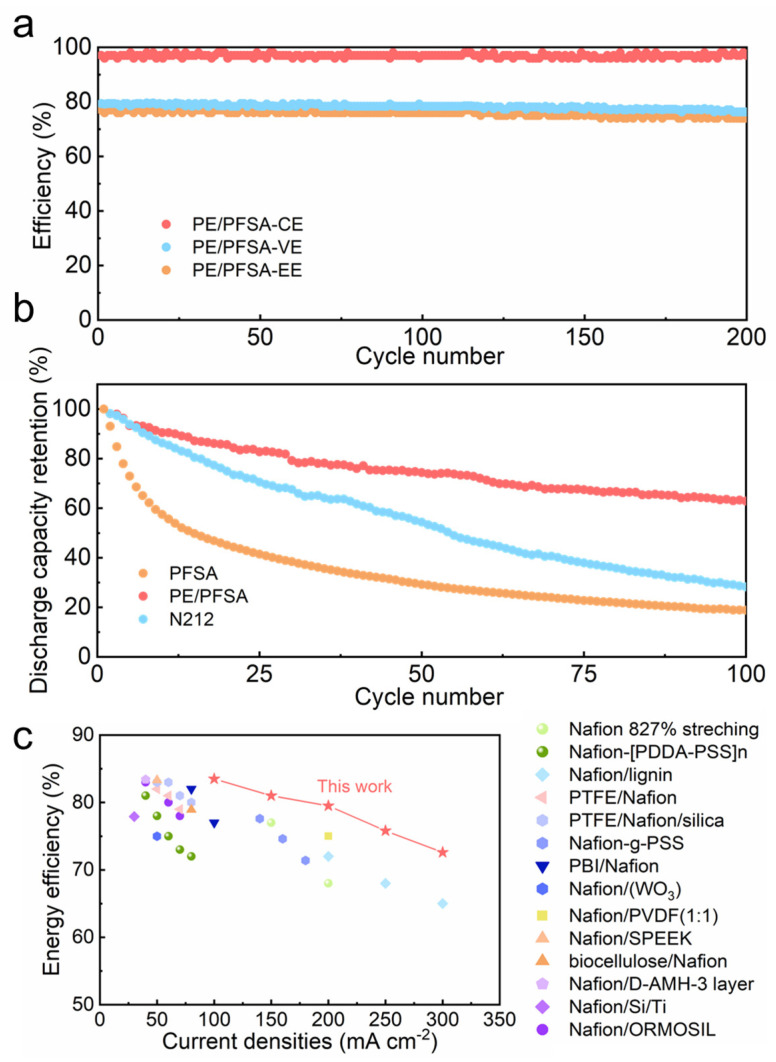
(**a**) Comparison of cycle efficiencies of the three membranes at 200 mA cm^−2^; (**b**) discharge capacity retention of the pure PFSA, PE/PFSA composite, and Nafion212 membranes; and (**c**) EE comparison between PE/PFSA and other existing advanced membranes at different current densities [7,26,33,34,35,36,37,38,39,40,41,42,43,44].

To determine the long-term performance of membranes, a VFB with different membranes was operated at 200 mA cm^−2^ in conditions where there were flowing electrolytes and strong acid and corrosion characteristics. As illustrated in Figure 6a, there was no obvious descending tendency of efficiencies including CE, VE, and EE for a VFB cell with a PE/PFSA composite membrane over 200 cycles. This verified that the PE/PFSA composite membrane possessed outstanding chemical stability. Moreover, capacity is another important performance parameter, which represents the ability to output energy. When there is ion crossover and water transmission, the transformation of electrolyte composition inevitably occurs and further leads to the attenuation of capacity. As shown in Figure 6b, the VFB with the PE/PFSA composite membrane had a lower capacity decay rate compared to the Nafion212 membrane. As can be seen, the discharge capacity retention of PE/PFSA was 62.9% after 100 cycles, whereas those of the pure PFSA and Nafion212 membranes were 18.8% and 28.2%, respectively. This is consistent with the notion that the PE/PFSA composite membrane effectively hinders the penetration of vanadium ions. What’s more, the PE/PFSA composite membrane maintained good mechanical stability due to its lack of reduced tensile strength and elongation at break (Figure S4). In conclusion, the thin PE/PFSA composite membrane (~20 μm) demonstrated superior performance. In addition, compared with other previously reported membranes, PE/PFSA demonstrated a higher EE (Figure 6c).

From a financial perspective, the amount of PFSA was cut, so the cost of the composite membrane was greatly reduced. The content of PFSA resin in commercial Nafion212 membranes is 100 g/m^2^, while it is reduced to approximately 25 g/m^2^ in a 20 μm PE/PFSA membrane. In addition, the average price of PE is far lower than that of PFSA, so the total material cost of PE/PFSA membrane is one-quarter that of Nafion212. These results confirm that the prepared composite membrane can greatly reduce the cost and thus generate financial benefits.

## 4. Summary

In this work, a low-cost composite membrane (~20μm) for VFB was designed and successfully prepared via the blending method using cost-effective microporous polyolefin membrane as the support and PFSA exchange resin as the composite phase. The PE/PFSA composite membrane with a dense microstructure exhibited extremely good mechanical properties, excellent dimensional stability, a low vanadium ion permeating rate, and high ion selectivity. Such good properties are attributable to the continuity of PFSA resin and robust binding with a rigid PE support. In addition, the VFB cell equipped with the PE/PFSA composite membrane demonstrated an excellent performance. Its EE value reached 79.8% at 200 mA cm^−2^ and it exhibited good long-term cycling stability within 200 cycles. Meanwhile, the cost of the composite was significantly reduced to one-quarter that of Nafion212. All in all, the PE/PFSA composite membrane demonstrated the potential for widespread use and provided inspiration for low-cost membrane design for a flow battery system.

## Data Availability

Data is unavailable due to privacy.

## Supplementary Materials

The following supporting information can be downloaded at: https://www.mdpi.com/article/10.3390/membranes13030272/s1, Figure S1: SEM images of the original PE (a) and the pretreated PE (b); Figure S2: SEM image of PE at high magnification. Figure S3: (a–c) CE, VE and EE of VFBs with pure PFSA, PE/PFSA, incomplete filled PE/PFSA and Nafion212 membranes at current densities from 100 to 300 mA cm^-2^. Figure. S4: The mechanical properties of the membranes. Table S1: Semi-quantitative content of elements of PE and pretreated PE.
