# Supplementary material for: An Economical Composite Membrane with High Ion Selectivity for Vanadium Flow Batteries
Source: Membranes (Basel). 2023 Feb 24;13(3):272. doi: 10.3390/membranes13030272 (PMC10057319; doi:10.3390/membranes13030272)
Supplement: Supplementary file 1 [file membranes-13-00272-s001.zip › membranes-2188946-supplementary.pdf]

Supplementary Information

An economical composite membrane with high ion selectivity for vanadium flow batteries

Yue Zhang [1,2], Denghua Zhang [1,2], Chao Luan [3], Yifan Zhang [1,2], Wenjie Yu [3], Jianguo Liu [1,*], Chuanwei Yan [1]

[1] Institute of Metal Research, Chinese Academy of Sciences, Shenyang, China

[2] School of Materials Science and Engineering, University of Science and Technology of China

[3] College of Chemistry, Liaoning University, Shenyang, China

[*] Corresponding author.

E-mail addresses: jgliu@imr.ac.cn (Jianguo Liu)

1. The pretreatment procedure

The SEM was conducted to observe the structure and composition change pf PE during the pretreatment process. As shown in Figure. S1a, b, the original PE and the pretreated PE both demonstrate a typical dendritic structure with many pores and the fibers are not broken. There is not obvious structure change. Besides, according to the semi-quantitative results of EDS mapping (Table. S1), the content of S and O elements slightly increase.

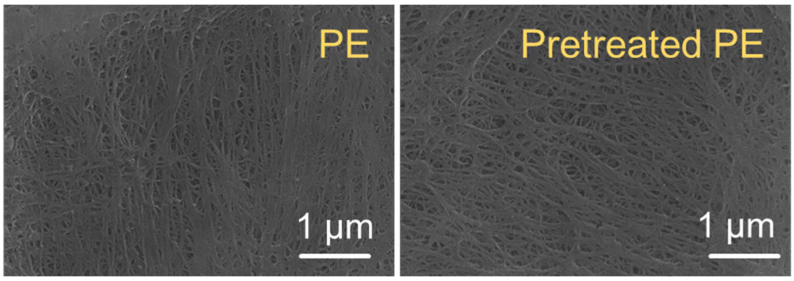

Figure S1 SEM images of the original PE (a) and the pretreated PE (b).

Table S1 Semi-quantitative content of elements of PE and pretreated PE.

samples	PE	Pretreated PE
C (wt%)	99.22	98.85
O (wt%)	0.71	0.83
S (wt%)	0.06	0.32
Total content (wt%)	100	100

2. The structure of PE

As shown in Figure. S2, it can be clearly visualized that the origin PE shows a typical dendritic network with many pores. The pore size is about 50-100 nm and the shapes of pores are diverse and unfixed. According to the specification parameters of PE, the density of pores is 42%.

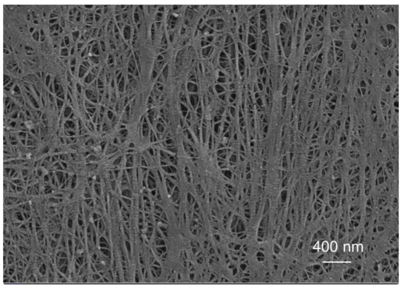

Figure S2 SEM image of PE at high magnification.

3. The unsuccessful experiment

For the membrane named incomplete filled PE/PFSA, the pores of PE are not completely filled and the membrane is not completely transparent on the macro level. As shown in the Figure. S3a, the CE of the incomplete filled PE/PFSA membrane is lower than that of the PE/PFSA and Nafion212. Because vanadium ions pass through the pores that are not completely filled in PE which increases the electrolyte crossing. Besides, the VE of the incomplete filled PE/PFSA membrane is the lowest among these membranes (Figure. S3b). Due to the introduction of insulated PE substrate, its conductivity is reduced. Besides the incomplete filling of PFSA resin, the number of dead-ends of PFSA conductive polymer is increased, which hinders the proton transfer. More importantly, the EE value is very low because of the low CE and VE (Figure. S3c), the incomplete filled PE/PFSA membrane exhibits

extremely poor performance.

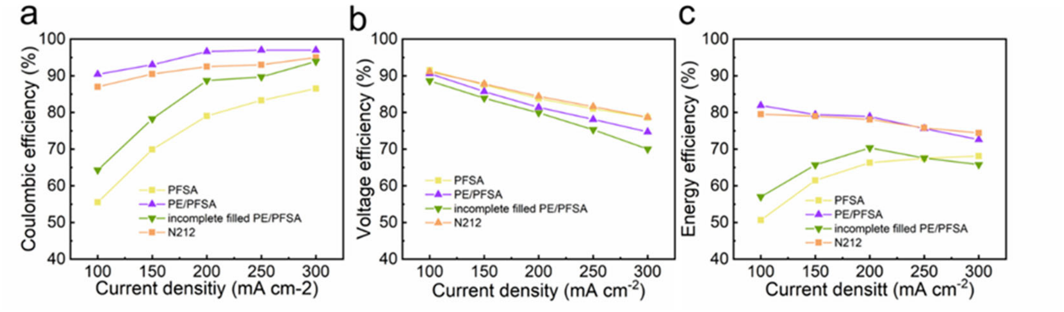

Figure S3 (a-c) CE, VE and EE of VFBs with pure PFSA, PE/PFSA, incomplete filled PE/PFSA and Nafion212 membranes at current densities from 100 to 300 mA cm^{-2}.

4. The mechanical properties of the PE/PFSA composite membrane after prolonged contact with a VO^{2+} solution.

The membranes were firstly soaked in the electrolyte (1.65 M $VOSO_4$/3 M H_2SO_4) at 60 °C for 5 d, then were washed several times and dried at 80 °C for 3 h. The treated PE/PFSA and N212 were named soaked PE/PFSA and soaked N212. As shown in Figure. S4, the tensile strength and elongation at break of the PE/PFSA composite membrane do not decrease. Besides, the elongation at break of the Nafion212 obviously decreases. Therefore, the PE/PFSA composite membrane possess a good chemical stability due to it maintenance of mechanical properties.

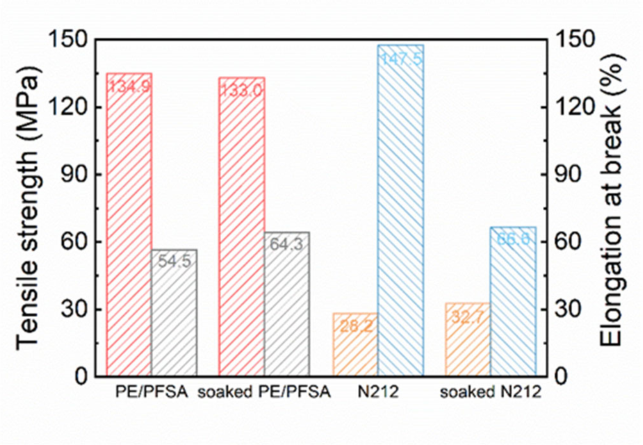

Figure S4 The mechanical properties of the membranes.
